# Patient-Reported Outcome Measures may optimize shared decision-making for cancer risk management in BRCA mutation carriers

**DOI:** 10.1007/s12282-019-01033-7

**Published:** 2019-12-12

**Authors:** L. S. E. van Egdom, M. A. de Kock, I. Apon, M. A. M. Mureau, C. Verhoef, J. A. Hazelzet, L. B. Koppert

**Affiliations:** 1 Department of Surgical Oncology, RG-228, Erasmus MC Cancer Institute, University Medical Centre Rotterdam, P.O. 2040, 3000 CA Rotterdam, The Netherlands; 2 Department of Public Health, Erasmus MC University Medical Centre Rotterdam, Rotterdam, The Netherlands; 3 Department of Plastic and Reconstructive Surgery, Erasmus MC Cancer Institute, University Medical Centre Rotterdam, Rotterdam, The Netherlands

**Keywords:** BRCA mutation carriers, Breast cancer risk management, Patient-reported outcomes, Shared-decision making

## Abstract

**Purpose:**

The aim of this study was to compare patient-reported outcomes (PROs) of BRCA1/2 mutation carriers, either after bilateral prophylactic mastectomy (BPM) or during breast surveillance, to improve shared decision-making in their cancer risk management.

**Methods:**

Unaffected *BRCA1/2* mutation carriers at least one year after BPM followed by immediate breast reconstruction (BPM-IBR) or one year under surveillance were eligible. After informed consent, the Hospital Anxiety and Depression Scale (HADS) and BREAST-Q were administered and compared between the different strategies. PROs were also compared to available normative data.

**Results:**

Ninety-six participants were analyzed in this study and showed significant differences between strategies in age, age at genetic testing, and time since BPM or starting breast surveillance. All HADS scores were below 8 suggesting no signs of anxiety or depression in both groups. Higher mean ‘Q-physical well-being’ scores were reported by the surveillance group (81.78 [CI 76.99–86.57]) than the BPM group (76.96 [CI 73.16 – 80.75]; *p* = 0.011). Overall, for both questionnaires better scores were seen when compared to age-matched normative data.

**Conclusions:**

No signs of anxiety or depression were seen in the surveillance or BPM-IBR group. Slightly better mean BREAST-Q scores were seen for the surveillance group in comparison to BPM-IBR, except for ‘Q-psychological well-being’. The difference in ‘Q-physical well-being’ was significantly worse for BPM-IBR. Approaches to obtain longitudinal PROs and reference values should be explored in the future, which could add value to shared decision-making in regards to breast cancer risk management in this specific patient population.

## Introduction

A woman’s lifetime risk of developing breast cancer is greatly increased when she inherits a *BRCA1* or *BRCA2* gene mutation. While the general population has a lifetime risk of 12% [1], *BRCA1* and *BRCA2* mutation carriers have a cumulative breast cancer risk of, respectively, 72% and 69% [2] till 80 years of age.

Breast cancer risk management for *BRCA1/2* mutation carriers encompass the possibility of intensive breast surveillance aimed at early detection, or bilateral prophylactic mastectomy (BPM). BPM has shown a risk reduction up to 95% [3-7] and is associated with decreased general and cancer-related distress [8, 9]. As BPM is a major, elective and irreversible procedure, however, it is also associated with a negative impact on health-related quality of life (HRQoL) outcomes such as body image, psychosocial-, psychosexual-, and physical well-being [8, 10, 11].

The alternative is intensive breast surveillance, consisting of annually alternating mammography and breast MRI, and semi-annual clinical breast examination commencing at 25 years of age [12]. Carriers who choose surveillance might have fewer problems with body image in the psychosocial- and psychosexual area, but will be confronted with difficulties concerning cancer-related distress and the risk of breast cancer [13].

Since BPM, either followed by immediate breast reconstruction (BPM-IBR) or not, and surveillance are both validated options with high survival rates [14], the choice between them is dependent on the individual woman’s preferences. To facilitate decision-making, it is important to fully explain the pros and cons of both options, especially when considering preference-based care for which there exists more than one clinically appropriate treatment option [15]. Therefore, women considering BPM(-IBR) should be informed about the impact of prophylactic surgery on not only survival and the risk of cancer but on the expected HRQoL outcomes as well [8, 15-18].

According to value-based healthcare principles, these HRQoL outcomes can be both provider-reported as well as patient-reported outcomes (PROs). Since PROs are direct assessments from patients, typically collected through validated questionnaires (i.e., PROMs = patient-reported outcome measurements), they reflect patients’ quality of life or functional status. PRO data is incredibly valuable to get insight into long-term HRQoL and can be used as a guide for *BRCA1/2* mutation carriers in their decision-making process in regard to their breast cancer risk management. However, little is known about PROs following the choice for either BPM or surveillance in *BRCA1/2* mutation carriers.

It was hypothesized that PROs differ between women choosing BPM(-IBR) and women opting for breast surveillance. This study aimed to compare PROs between *BRCA1/2* mutation carriers following their choice for either BPM-IBR or breast surveillance.

## Methods

### Study population

A total of 96 unaffected *BRCA1/2* mutation carriers, diagnosed at the Academic Breast Cancer Centre of the Erasmus MC between 2014 and 2017, were included. Female *BRCA1/2* mutation carriers, aged over 18 years and with an adequate understanding of the Dutch language, were deemed eligible. Mutation carriers who were at least 1 year post-BPM-IBR (autologous or implants) were identified from the electronic health records using operation and diagnosis codes. Mutation carriers scheduled for at least one year of breast surveillance were approached at the surgical oncology outpatient clinic. Mutation carriers were asked to participate until at least 50 participants were enrolled in each group. Women with a past history of (in situ) breast cancer were excluded. Ethical approval was granted by the Institutional Review Board of the Erasmus Medical Centre, Rotterdam, The Netherlands (MEC-2018–1601).

### Procedures

In this cross-sectional study, medical records were retrospectively reviewed to collect the following data: *BRCA1/2* mutation status and date of genetic testing, age at genetic testing, family history of breast cancer, comorbidities, smoking status, family status, time since starting breast surveillance, or time since surgery, and—if applicable—type of surgery performed. Missing data was handled by contacting the participant via telephone. For the BPM-IBR group, eligible women were recruited by telephone or mail. Women in the surveillance group were asked to participate at the outpatient clinic. Following informed consent, two PROM questionnaires were administered: the Hospital Anxiety and Depression Scale (HADS) [19] and the BREAST-Q version 1.0 (pre-mastectomy module for the surveillance group and the post-reconstruction module for BPM-IBR) [20]. Both PROMs were web-based questionnaires and administered through the software program “GemsTracker” [21], an online system for distributing and collecting surveys. If the questionnaires remained uncompleted, a weekly reminder was sent by the system. If patients had not responded in four weeks, participants were contacted by telephone and asked to complete the questionnaires. PROM scores were calculated according to the questionnaires’ scoring manuals.

### Statistical analysis

All analyses were performed using Statistical Package for Social Sciences (SPSS), Version 24.0 (IBM Corporation, Armonk, NY, USA). Baseline characteristics were compared between women who underwent BPM-IBR versus those who chose breast surveillance. Comparisons across both groups were made using the Mann–Whitney U test for continuous variables, and Fisher’s exact test or the Chi-squared test, as appropriate, for categorical variables. Two-sided *p* value < 0.05 was considered statistically significant. In addition, PROM scores were compared to normative scores [22-24].

## Results

### Study population

**Fig. 1:**
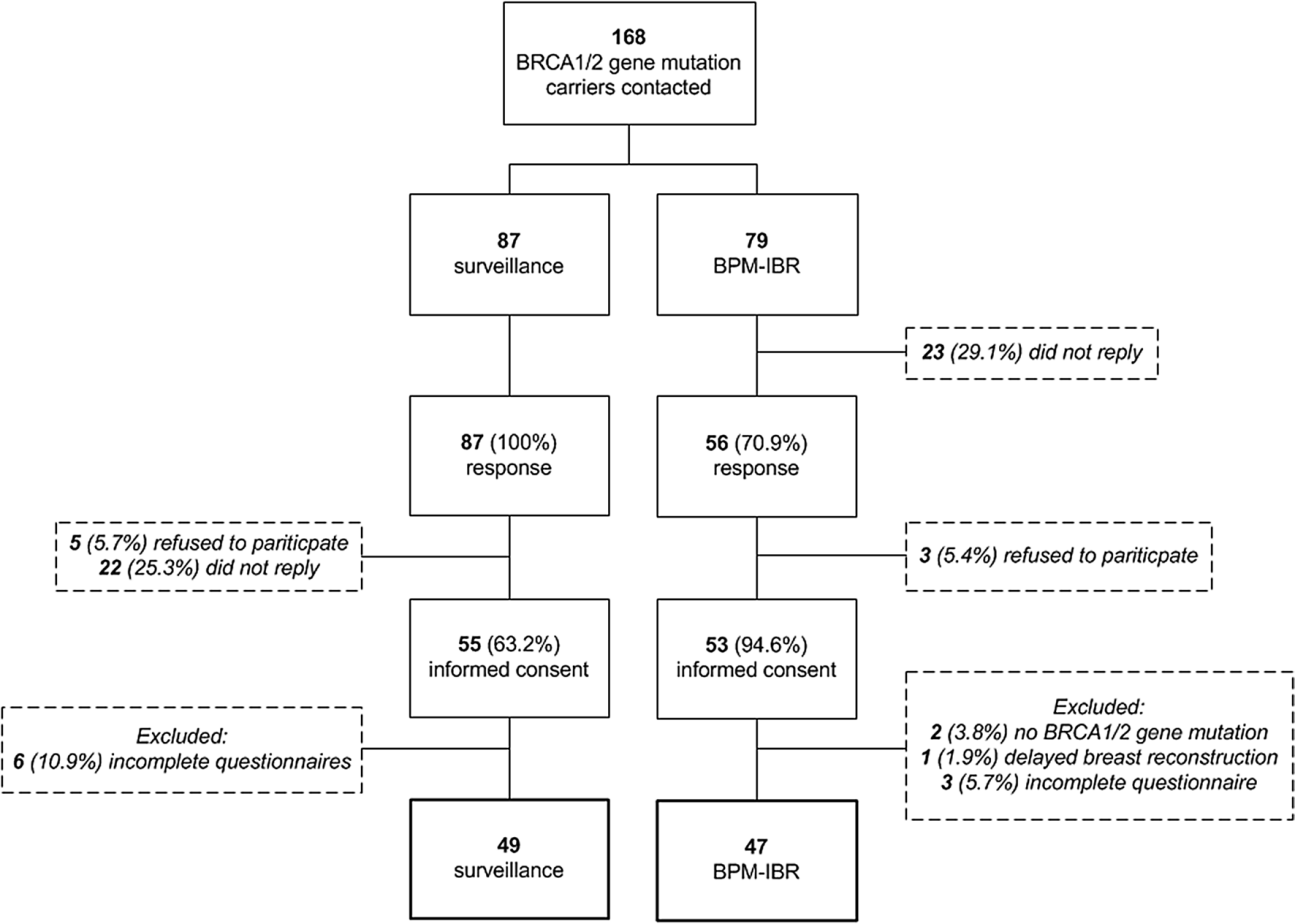
Flowchart of study selection process. *BPM-IBR*, bilateral prophylactic mastectomy followed by immediate breast reconstruction

Between October 2018 and May 2019, 168 women were contacted via telephone, mail or at the outpatient clinic (Fig. 1). Of the eligible participants, 143 (85%) women responded. Eight (5.6%) responders declined participation. Of the surveillance group, 22 (25.3%) responders did not reply despite verbal consent being obtained at the outpatient clinic. After informed consent, 55 (63.2%) women participated in the surveillance group and 53 (67%) in the BPM-IBR group. Three women were excluded from the BPM-IBR group: one woman underwent a delayed breast reconstruction and the other two due to the absence of a *BRCA1/2* gene mutation.

### Characteristics

A total of 96 participants were included for analysis: 47 BPM-IBR and 49 breast surveillance participants (Table 1). Statistically significant differences were seen between both groups in age at study enrollment, age at genetic testing, and time since surveillance start or since BPM-IBR. Overall, the study population was relatively young: 43% of the surveillance group and 45% of the BPM-IBR group were aged below 35 years. Approximately 60% of both groups had a positive family history for breast cancer in two or more relatives. Risk-reducing bilateral salpingo-oophorectomy (RRSO) or prophylactic tubectomy were performed in, respectively, 45% and 11% of the study population.

**Table 1:** Characteristics of 96 *BRCA1/2* mutation carriers per type of cancer risk management, *n* (%)

	All (*n* = 96)	Surveillance (*n* = 49)	BPM-IBR (*n* = 47)	*p* value
Mean (SD) age (years)^§^	42.4 (10.7)	44.5 (12.0)	40.2 (8.8)	0.046
Mean (SD) age (years) at genetic testing^§^	36.6 (10.3)	38.7 (10.6)	34.3 (9.6)	0.039
*Mutation type*^¥^				0.969
*BRCA1*	57 (59)	29 (59)	28 (60)	
*BRCA2*	39 (41)	20(41)	19 (40)	
Mean (SD) age (years) at start cancer risk management^§^	37.9 (9.8)	38.7 (10.7)	37.1 (8.7)	0.447
Mean (SD) time (years) since start of cancer risk management^§^	4.7 (3.7)	6.1 (4.7)	3.1 (1.2)	0.002
*Family *history^¥^				0.723
0	13 (14)	7 (14)	6 (13)	
1	24 (25)	10 (20)	14 (30)	
≥ 2	57 (59)	30 (61)	29 (62)	
Unknown	2 (2)	2 (4)	.0	
*First degree family history*^¥^				0.176
0	66 (69)	30 (61)	36 (77)	
1	28 (29)	17 (35)	11 (23)	
≥ 2	.0	.0	.0	
Unknown	2 (2)	2 (4)	.0	
*Second degree family history*^¥^				0.229
0	63 (66)	32 (65)	31 (66)	
1	28 (29)	15 (31)	13 (28)	
≥ 2	.0	.0	3 (6)	
Unknown	2 (2)	2 (4)	.0	
*Third degree family history*^¥^				0.617
0	32 (33)	16 (33)	16 (34)	
1	31 (32)	14 (29)	17 (36)	
> 2	31 (32)	17 (35)	14 (30)	
Unknown	2 (2)	2 (4)	.0	
*Marital status*^¥^				0.079
Single	8 (8)	1 (2)	7 (15)	
Relationship	21 (22)	11 (22)	10 (21)	
Married	58 (60)	30 (61)	28 (60)	
Unknown	9 (9)	7 (14)	2 (4)	
Parity, mean (SD)^§^	1.4 (1.0)	1.4 (0.9)	1.5 (1.07)	0.461
*Ovarian status*^¥^				0.147
In situ	31 (32)	16 (33)	15 (32)	
RRSO	45 (47)	27 (55)	18 (38)	
Tubectomy	11 (11)	3 (6)	8 (17)	
Unknown	9 (9)	3 (6)	6 (13)	
*Smoking status*^¥^				0.910
Yes	9 (9)	4 (8)	5 (11)	
No	73 (76)	31 (63)	42 (89)	
Unknown	14 (15)	14 (29)	.0	

*BPM-IBR* bilateral prophylactic mastectomy followed by immediate breast reconstruction, *RRSO* risk-reducing bilateral salpingo-oophorectomy;

^¥^Chi-squared test

^§^Mann–Whitney *U* test

### PROMs

Table 2 gives an overview of the PROM scores. For both groups, all individual HADS scores were below eight, which was defined as the upper limit of normal [22]. Slightly better mean BREAST-Q scores were seen in the surveillance group as compared to the BPM-IBR group, except for the domain ‘Q-psychological well-being’. In contrast, only the difference in ‘Q-physical well-being’ was significantly higher in the surveillance group (81.78; CI 76.99–86.57) than the BPM-IBR group (76.96; CI 73.16–80.75; *p* = 0.011).

**Table 2:** PROM scores of 96 *BRCA1/2* mutation carriers per type of cancer risk management, mean (95% CI)

	All (*n* = 96)	Surveillance (*n* = 49)	BPM-IBR (*n* = 47)	*p* value^¥^
	Mean (95% CI)	Mean (95% CI)	Mean (95% CI)	
*HADS°*				
Anxiety scale	5.36 (4.62–6.09)	5.47 (4.30–6.63)	5.26 (4.30–6.21)	0.691
Depression scale	2.40 (1.80–3.00)	2.51 (1.66–3.36)	2.30 (1.42–3.18)	0.591
*BREAST-Q*^*§*^				
Satisfaction with breasts	68.96 (65.09–72.82)	71.51 (65.56–77.47)	66.51 (61.42–71.60)	0.304
Psychosocial well-being	74.08 (70.14–78.01)	70.78 (65.17–76.38)	77.23 (71.67–82.80)	0.143
Physical well-being chest	79.32 (76.29–82.34)	81.78 (76.99–86.57)	76.96 (73.16–80.75)	0.011
Sexual well-being	61.53 (57.87–65.20)	62.82 (57.24–68.41)	60.30 (55.33–65.27)	0.644

HADS index value: scale from 0–21; BREAST-Q scale from 0 to 100

*BPM-IBR* bilateral prophylactic mastectomy followed by immediate breast reconstruction, *HADS* Hospital Anxiety and Depression Scale

°Higher scores represent lower quality

^§^Higher scores represent higher quality

^¥^Mann–Whitney *U* test

**Fig. 2:**
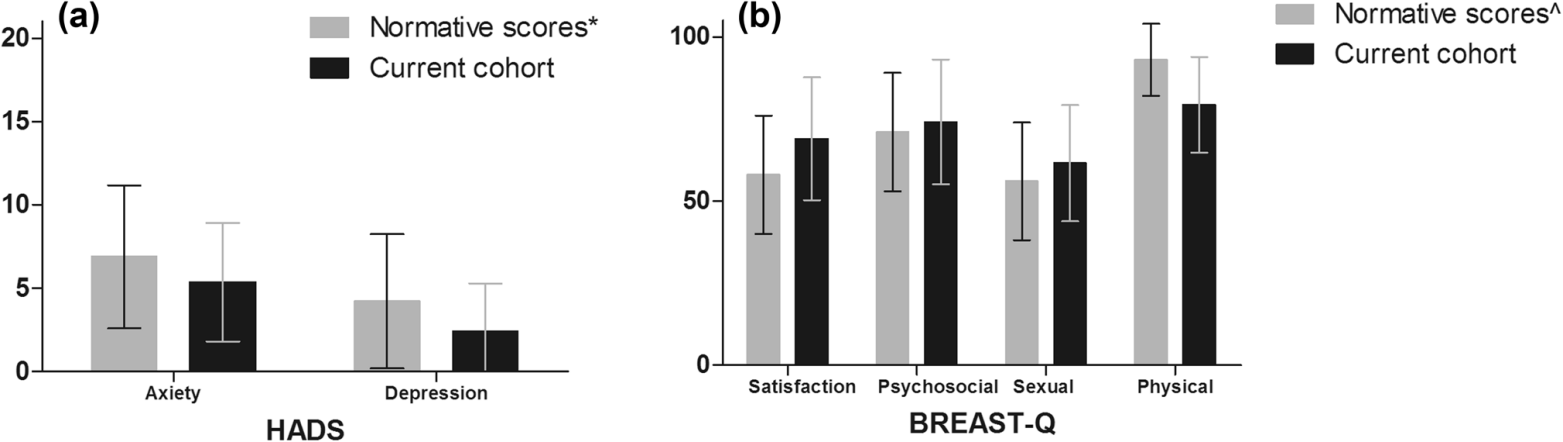
Comparison of PROM scores with normative scores. **a** HADS survey scores versus normative scores [22]. Mean scores with standard deviations (error bars) for HADS scores. HADS index value: scale from 0–21. Higher scores represent lower quality. *Normative scores as based on 486 patients (anxiety subscale) and 489 patients (depression subscale) [22], presented by gender and age (i.e., female and 5-year age group 40–44 years). **b** BREAST-Q survey scores versus normative reconstructive scores [23]. Mean scores with standard deviations (error bars) for Q-scores. BREAST-Q scale from 0 to 100. Higher scores represent better functioning. ^Normative scores as based on 1201 participants of the general population [23]

Obtained HADS scores were compared to normative data [22], demonstrating lower scores on both the anxiety and the depression scale in both groups (Fig. 2a). As the mean age of our cohort was 42.4 years, normative data of the female age category 40–44 years was used for comparison. The normative data of the preoperative reconstruction module was used for the comparison with BREAST-Q scores of both groups in our cohort [23, 24]. PROMs were comparable to normative scores of the BREAST-Q except for the ‘Q-physical well-being’ scale, which showed lower scores in the current cohort (Fig. 2b).

## Discussion

*BRCA1/2* mutation carriers are faced with complex decisions within breast (and ovary) cancer risk management. Insights into not only cancer risk but also into HRQoL or daily functioning as a result of these decisions could improve the shared decision-making process and ultimately the care delivered. Therefore, this study aimed to obtain and evaluate PROs in *BRCA1/2* mutation carriers according to their choice of breast cancer risk management (BPM-IBR versus breast surveillance).

The PROMs in this study have succeeded in providing valuable insights into HRQoL in *BRCA1/2* mutation carriers, in both the BPM-IBR and the breast surveillance group. The interpretation of these data was done both separately and in comparison to available normative data [22–24].

HADS demonstrated no scores outside normal cut-off values on the two scales. Moreover, mean scores observed for both groups were quite similar and all reported scores were below the upper limit. These observations indicate that none of the mutation carriers in the present study reported anxiety or depression that reached clinically relevant levels. In addition, no significant differences in anxiety or depression outcomes were observed between women in the surveillance group and the BPM-IBR group.

Overall, slightly better BREAST-Q scores were seen for the surveillance group compared to BPM-IBR. The surveillance group scored lower on ‘Q-psychological well-being’, albeit not statistically significant. This difference was expected since previous studies have already shown elevated levels of psychological distress in women at increased risk of developing breast cancer [8, 13]. Only the difference in ‘Q-physical well-being’ was statistically significant, which can be explained by the surgical procedure these women have undergone. However, it has been acknowledged that not only the statistical significance of the differences in QoL questionnaires is important but the clinical relevance of them as well [25]. Although there is no consensus yet on clinically relevant BREAST-Q scores, it is generally accepted that a difference of 5 points should be considered as a small clinical difference, 10 points as moderate, and 20 points as a very clinically important difference [26]. There was a difference of 5–10 points for all BREAST-Q modules except for ‘Q-sexual well-being’, which suggests a small clinical difference between both groups. PROs should be collected longitudinally to evaluate the clinical differences in PROM scores over time within both groups.

Of all BREAST-Q subscales, the lowest scores were reported for ‘Q-sexual well-being’ by both BPM-IBR and breast surveillance women. Previous studies have shown that breast cancer surgery may have a negative impact on sexual health [27, 28]. The low ‘Q-sexual well-being’ scores might also be explained by the high proportion of women with a risk-reducing ovarian cancer intervention (RRSO or tubectomy). Since RRSO substantially decreases the levels of estrogen and testosterone, it has an effect on quality of life and sexual functioning, among other domains, at an early age [29, 30]. However, we also compared mean ‘Q-sexual well-being’ scores between women with and without RRSO/tubectomy and found slightly higher mean scores in the RRSO/tubectomy group (i.e., 65.04 [60.01–70.07] and 55.90 [49.43–62.38], respectively). This emphasizes our rationale of the impact that breast surgery can have on a woman’s sexual health, which is in line with our previous publication also showing low ‘Q-sexual well-being’ scores in surgical treated breast cancer patients (without a *BRCA1/2* mutation) [31]. Also noteworthy is that only 33.9% of the women were treated with hormone replacement therapy (*n* = 8 in the BMP-IBR and *n* = 11 in the surveillance group) (data not shown).

Available normative data for the HADS were derived from the Epidemiology of Functional Disorders (EpiFunD) Study [22] and normative data for the BREAST-Q from the Army of Women community [23]. When comparing the PROM scores of our cohort with the normative data, one must take into account that the normative data were obtained in the United Kingdom (HADS) and the United States (BREAST-Q). Due to cultural differences between these countries and the Netherlands, this data does not entirely reflect normative scores for Dutch women. However, similar Q-scores were seen when comparing the current cohort with Dutch cohorts [27, 32]; i.e., overall better scores except for ‘Q-psychical well-being’. HADS scores were not available within these cohorts.

Significant differences in patient characteristics existed between both groups, suggesting a possible treatment indication bias. Available data on the impact of patient characteristics on a woman’s decision to undergo BPM vary. Most studies show that age at genetic testing does not significantly affect the choice for BPM [8, 13, 15, 33], which is in opposition to our findings. On the other hand, no significant differences in family history, ovarian status, marital status, and parity existed between both groups, in contrast to other studies showing that these factors *do* have a significant impact on the choice for BPM [10, 13, 33-35]. However, due to the retrospective design of this pilot study, baseline (anxiety) scores could not be obtained. Women may experience physical- and psychological trauma associated with being diagnosed with a *BRCA1/2* mutation, which will affect their HRQoL. Thus, changes in PROs before and after diagnosis are to be expected, which emphasizes the necessity of PRO collection at baseline.

The significant differences in age at study enrollment, age at genetic testing, and the time since BPM-IBR or starting breast surveillance could be explained by the manner in which women were selected. Eligible participants for the BPM-IBR group were found through a search in the electronic health record. The search was thereby limited by year of surgery, namely between 2014 and 2017. Gene mutation carriers scheduled for breast surveillance were asked to participate at the outpatient clinic. No limitations on patient inclusion was set for this group and could, therefore, be completed before 2014. Although the duration of the inclusion period was over 6 months and all *BRCA1/2* mutation carriers were scheduled for follow-up every 6 months during their surveillance, a potential selection bias could have been introduced. Moreover, the time since the start of cancer risk management significantly differed between both groups (6.1 years for surveillance versus 3.1 years for BPM-IBR, *p* = 0.002), and time since BMP-IBR was relatively short. Previous studies have shown that psychological outcomes as well as coping strategies change over time [8, 13] [13]. Coping strategies represent cognitive and behavioral efforts to deal with stressful encounters [36]. Effects of coping can differ depending on the duration and controllability of the stress factor. As women in our cohort did not have a history of breast cancer (consistently favorable results during their surveillance), long-term breast cancer-related distress might decrease as a consequence of ‘underestimating' their breast cancer risk [13]. This observation may be a possible explanation for the low distress and anxiety levels in our cohort. Another possible explanation for the low scores is potential selection bias, as the women who experienced increased levels of depression might have been less likely to participate.

We did not find that women in the BMP-IBR group were more likely to have a first-degree relative with a history of breast cancer (35% surveillance versus 23% BPM-IBR, *p* = 0.176), which is in contrast to what others have reported [33, 37].

Intuitively, it would seem that women with a *BRCA1* mutation would most likely be the ones to consider BPM as they have a higher breast cancer risk than *BRCA2* mutations [2]. Moreover, a previous study with 5,889 Dutch *BRCA1/*2 mutation carriers showed that, compared to breast surveillance, BPM was associated with lower mortality for *BRCA1* mutation carriers, whereas for *BRCA2* mutation carriers breast cancer-specific survival rates were similar between BPM and breast surveillance [38]. In our cohort, however, there were no differences in the percentage of *BRCA1* carriers in the BPM-IBR group compared to the surveillance group. The observations that BPM was associated with lower mortality rates than surveillance for *BRCA1* and similar breast cancer-specific survival for *BRCA2*, underscore the importance of counseling *BRCA1/2* mutation carriers on their choice between breast surveillance and BPM. Knowledge of patient-reported HRQoL outcomes can be valuable in facilitating this choice.

Limitations include the relatively small sample size and the retrospective study design. The power was limited due to the small study population. Longitudinal PRO collection and comparison with baseline PROM scores are needed when striving to showcase the influence of different risk management strategies [13, 15]. However, the retrospective evaluation of PROs does provide the necessary insight into (case-mix) factors possibly associated to PROs, and their inclusion for predictive modeling.

Multiple PROM instruments are available nowadays. Only two questionnaires were selected in this study. HADS was chosen since it is a short questionnaire and the most extensively validated scale for screening emotional distress in cancer patients [39], while BREAST-Q was chosen since it is a validated breast-specific instrument that is used worldwide. Razdan et al. [15] evaluated PROs after BPM and concluded that generic instruments were not sensitive enough to measure physical and mental changes related to specifically BPM, either followed by (immediate) breast reconstruction or not. The use of a breast-specific instrument (e.g., BREAST-Q) was recommended. We support this recommendation combined with the standardization of PROMs, since this will provide results that are comparable with other similar studies.

Several initiatives of longitudinal PRO collection in breast cancer patients have proven to be helpful in daily practice and are appreciated by both patients and providers [40, 41]. The present study provides a first insight into PROs in *BRCA1/2* mutation carriers following their choice for either breast surveillance or BPM-IBR. Collected PROs can serve to pave the way for the implementation of a value-based healthcare strategy among future *BRCA1*/*2* mutation carriers at the outpatient clinic. Interpretability of the important differences in PRO(M)s is the cornerstone to its successful use in individual clinical care, comparative effectiveness research, and regulatory efforts. Knowledge about differences in HRQoL outcomes between BPM and surveillance can be used to facilitate shared decision-making. Informing *BRCA1/2* mutation carriers about both positive and negative consequences of either BPM-IBR or breast surveillance is of great importance for building up realistic expectations [9]. Measuring PROs in *BRCA1/2* mutation carriers from gene mutation diagnosis to the subsequent trajectory has the potential to monitor and detect changes in psychosocial or physical problems over time. Reference PROM scores for the different strategies are then essential for the use of PROs at the outpatient clinic to personalize and improve the care delivered. Large multicenter initiatives and prospective PRO collections are, therefore, needed to obtain (and narrow down) these reference scores. Such an initiative is currently pending at our institution.

## Conclusion

Patient-reported HRQoL outcomes were evaluated in unaffected BRCA1/2 mutation carriers who underwent either breast surveillance or BPM-IBR. No signs of anxiety or depression were seen in both groups. Slightly better mean BREAST-Q scores were seen for the surveillance group in comparison to BPM-IBR except for ‘Q-psychological well-being’; the difference in ‘Q-physical well-being’ was significantly worse for BPM-IBR. A first step was made towards value-based healthcare for BRCA1/2 mutation carriers. Future possibilities should be explored to obtain reference PROM values, which could add value to the shared decision-making process in regard to cancer risk management in this specific population.

## Abbreviations

PRO(M): Patient-reported outcome (measures)
BRCA: The BReast CAncer gene
BPM: Bilateral prophylactic mastectomy
IBR: Immediate breast reconstruction
HADS: Hospital Anxiety and Depression Scale
CI: Confidence internal
HRQoL: Health-related quality of life
RRSO: Risk-reducing salpingo-oophorectomy
